# Suicide death and hospital-treated suicidal behaviour in asylum seekers in the Netherlands: a national registry-based study

**DOI:** 10.1186/1471-2458-11-484

**Published:** 2011-06-21

**Authors:** Simone Goosen, Anton E Kunst, Karien Stronks, Irene EA van Oostrum, Daan G Uitenbroek, Ad JFM Kerkhof

**Affiliations:** 1 Netherlands Association for Community Health Services, P.O. Box 85300, 3508 AH Utrecht, the Netherlands; 2 Department of Public Health, Academic Medical Center, University of Amsterdam, P.O. Box 22660, 1100 DD Amsterdam, the Netherlands; 3 Quantitative Skills, Consultancy for Research and Statistics, Lieven de Keylaan 7, 1222 LC Hilversum, the Netherlands; 4 Public Health Service, P.O. Box 2200, 1000 CE Amsterdam, the Netherlands; 5 Department of Clinical Psychology, EMGO+ Institute, VU University, Van der Boechorststraat 1, 1081 BT, Amsterdam, the Netherlands

**Keywords:** suicide, suicidal behaviour, migrants, asylum seekers, refugees

## Abstract

**Background:**

Several suicide and suicidal behaviour risk factors are highly prevalent in asylum seekers, but there is little insight into the suicide death rate and the suicidal behaviour incidence in this population. The main objective of this study is to assess the burden of suicide and hospital-treated non-fatal suicidal behaviour in asylum seekers in the Netherlands and to identify factors that could guide prevention.

**Methods:**

We obtained data on cases of suicide and suicidal behaviour from all asylum seeker reception centres in the Netherlands (period 2002-2007, age 15+). The suicide death rates in this population and in subgroups by sex, age and region of origin were compared with the rate in the Dutch population; the rates of hospital-treated suicidal behaviour were compared with that in the population of The Hague using indirect age group standardization.

**Results:**

The study included 35 suicide deaths and 290 cases of hospital-treated suicidal behaviour. The suicide death rate and the incidence of hospital-treated suicidal behaviour differed between subgroups by sex and region of origin. For male asylum seekers, the suicide death rate was higher than that of the Dutch population (N = 32; RR = 2.0, 95%CI 1.37-2.83). No difference was found between suicide mortality in female asylum seekers and in the female general population of the Netherlands (N = 3; RR = 0.73; 95%CI 0.15-2.07). The incidence of hospital-treated suicidal behaviour was high in comparison with the population of The Hague for males and females from Europe and the Middle East/South West Asia, and low for males and females from Africa. Health professionals knew about mental health problems prior to the suicidal behaviour for 80% of the hospital-treated suicidal behaviour cases in asylum seekers.

**Conclusions:**

In this study the suicide death rate was higher in male asylum seekers than in males in the reference population. The incidence of hospital-treated suicidal behaviour was higher in several subgroups of asylum seekers than that in the reference population. We conclude that measures to prevent suicide and suicidal behaviour among asylum seekers in the Netherlands are indicated.

## Background

In 2008 an estimated 383 000 asylum applications were recorded in 51 Western countries, including most European countries, the USA and Canada [1]. Asylum seekers are people who have left their country of origin, applied for protection as a refugee in another country, and are awaiting a decision on their application [2].

Recent reviews quite clearly identify mental disorders, such as depression, schizophrenia, substance use disorder, personality disorder and comorbid anxiety disorder, as the most prominent risk factors for suicide [3-5]. Other risk factors for suicide are traumatic life events and psychosocial crisis [3]. The stress-diathesis model for suicides, however, states that suicide is never the consequence of one single cause or stressor but also requires a predisposition for suicidal ideation, with psychiatric illness and psychosocial crises as proximal stressors [3]. This means that most people will not have suicide ideation, even in very difficult circumstances. Risk factors for non-fatal suicidal behaviour include the number of stressful life events, family disruption, lack of social support, low income, unemployment, previous traumatic experiences [5-7]. In people presenting with first-ever suicidal behaviours, the prevalence of psychiatric disorders may be rather low, whereas socio-economic deprivation (low education, low income, unemployment, poverty and divorce) is much more prevalent [6]. A recent general review showed a moderate association between post-traumatic stress disorder (PTSD) and suicidal ideation, but no evidence for a link between PTSD and completed suicide [8].

It is known that several suicide and suicidal behaviour risk factors are highly prevalent in asylum seekers [9-13]. However, data about the suicide death rate and the incidence of non-fatal suicidal behaviour among asylum seekers are very limited. To our knowledge, the only study that reports rates for suicide death and suicidal behaviour specifically for asylum seekers was conducted in Denmark (2001-2003), but it included only three cases of suicide [14]. A UK study into suicide and self-harm among asylum seekers did not publish any rates, and concluded that the number of asylum seekers who die from suicide or have suicidal behaviour is still unknown [15]. A study into the influence of country of birth on the risk of suicidal behaviour - conducted in Sweden - shows large differences between sexes and between countries/regions of origin, but does not provide specific data on asylum seekers [7]. On the basis of differences in the suicide death rate and the suicidal behaviour incidence worldwide [3], we expected that suicide and suicidal behaviour risks are not evenly distributed between subgroups of asylum seekers.

Insight into the burden of suicide death and suicidal behaviour is necessary in order to determine whether extra preventive efforts are required for asylum seekers; such insight could also provide directions for prevention [16,17]. The main objective of the current study was to assess the burden of suicide and hospital-treated suicidal behaviour in asylum seekers in the Netherlands and to identify factors that could guide prevention.

The specific steps taken were as follows:

• to assess the suicide mortality rate and the incidence of hospital-treated suicidal behaviour in asylum seekers, and compare it to the Dutch population and the population of The Hague;

• to assess the extent to which health staff were aware of the existence of mental health problems in asylum seekers prior to the date of hospital-treated suicidal behaviour and the extent to which these cases were under mental health treatment prior to the date of the hospital-treated suicidal behaviour;

• to determine which methods were used by cases of suicide death and of hospital-treated suicidal behaviour and what stressors were reported in cases of hospital-treated suicidal behaviour.

## Methods

### Context of the study

Asylum seekers in the Netherlands are housed in residential reception centres, managed by the Central Agency for the Reception of Asylum Seekers (COA). Asylum seekers are free to leave the centres and are allowed to work for a limited number of weeks per year. They are entitled to full access to health care. Up until 2008, COA contracted a health insurance company to arrange curative health care for asylum seekers through access to mainstream health services, including inpatient and outpatient mental health services. COA also contracted local public health services (GGDs) to provide preventive health services and specific nurse practitioner services, collectively called Community Health Services for Asylum Seekers (MOA). MOA nurse practitioners working at the reception centres provided a bridge function between the asylum seeker and regular health care. Asylum seekers with mental health problems could be offered up to five consultations with a public health doctor specialized in refugee health care to clarify any mental health or other health problems and needs. Asylum seekers could be referred, with or without these consultations, to the mental health service and/or offered preventive interventions. Since January 2009 the health system for asylum seekers in the Netherlands has changed.

### Data sources

We collected data on suicide deaths and hospital-treated suicidal behaviour cases that took place among asylum seekers living in reception centres in the Netherlands from 2002-2007. Under a national protocol, public health doctors and nurses at the MOA were required to complete a standard form for every case of death (general death notification form) and suicidal behaviour [18]. Neither the protocol nor the form gave a definition of suicidal behaviour. As it is difficult to determine in self-injury cases whether there was an intent to die [19], this study does not further differentiate self-injury cases. In order to limit the study to cases with considerable injury, only cases of suicidal behaviour treated at a hospital were included. This categorisation was made on the basis of a question on the form asking whether and where the asylum seeker was treated after the suicidal behaviour.

Repeated suicidal behaviour cases were included in the database as separate events if these were reported on different forms. Data about suicide deaths were extracted from the general asylum seeker mortality database [18]. Statistics Netherlands (CBS) coded the cause of death using the ICD-10. We included all cases allocated ICD-codes X60-X84 or Y87.0 [18].

We grouped countries of origin into regions according to the United Nations High Commissioner for Refugees (UNHCR) classification [2]. Some regions were combined because of small numbers; data for regions with very small populations were not presented. The regions are as follows (in brackets the abbreviations used and the two most frequently encountered countries): West, Central, Southern Africa (WCS Africa; Angola, Democratic Republic of Congo), North, East, Horn of Africa (NEH Africa; Somalia, Sudan), Central, East and Southern Europe (CES Europe; Azerbaijan, former Yugoslavian countries), Middle East/South West Asia (ME/SW Asia; Afghanistan, Iraq).

The suicidal behaviour notification form included open questions on medical history, stressors and methods used. Classification of these variables was performed by SG and AK, who also classified the methods used in suicide deaths. The form also contained dichotomous questions on whether the health professional knew of any mental health problems in the asylum seeker prior to the suicidal behaviour (without any further specification) and whether the asylum was using mental health treatment prior to the suicidal behaviour. No further specification of mental health problems and treatment were included as the questions were included with an exploratory objective.

The COA (box 1) provided data on the number of asylum seekers by sex, age group and country of origin on every first day of the month in the study period. These were used to calculate estimates of the person years spent in reception facilities. These data are considered good estimates for the denominator.

Comparison of the suicide death rate in asylum seekers with that of the general population of the Netherlands was performed using 2002-2007 data from the CBS national mortality register [20]. Suicidal behaviour data were compared with data from a study in the Dutch city of The Hague, as no national data were available. The study in The Hague covered all cases of hospital-treated suicidal behaviour cases in 2002-2004, based on data from the emergency departments of the city's four general hospitals [21].

### Statistical analysis

The rates used - the suicide mortality rate and the incidence rate of hospital-treated suicidal behaviour - were calculated for the population aged 15 years and older per 100 000 person-years. The numerators were the reported number of suicide deaths and cases of hospital-treated suicidal behaviour. Person-years at risk were calculated on the basis of the occupancy numbers for asylum seeker centres on the first day of each month during the study period.

Rate ratios were calculated using indirect standardization [22]. The observed numbers of suicide death and hospital-treated suicidal behaviour were compared with the expected numbers based on the rates for the reference populations, specifically for age and sex. The expected numbers were obtained by multiplying the person-years at risk in each category by the age and sex-specific suicide death rates in the Netherlands [20], and hospital-treated suicidal behaviour incidence rates in The Hague respectively [21]. Finally, the observed/expected (O/E) ratios and the 95% confidence intervals (CIs) were calculated using Byar's approximation of the exact interval for the Poisson distributed variables. This approximation is accurate even with small numbers [23].

To make comparisons across subgroups of asylum seekers, with regards to the percentage of people with hospital-treated suicidal behaviour known to have had mental health problems and the percentage receiving treatment for these problems, we calculated prevalence rate ratios and 95%CI with SISA's t-test procedure [24].

## Results

### Burden of suicide death and hospital-treated suicidal behaviour

In total 35 cases of death from suicide were recorded (table 1), resulting in a suicide mortality rate of 17.5/100 000/year. Mortality was much higher in males than females (risk ratio = 7.3, 95% CI = 2.2-23.7). Suicide mortality was more common in male asylum seekers than in males in the general population in the Netherlands. No difference was found between suicide mortality in female asylum seekers and in the female general population of the Netherlands (table 1). Compared to the Dutch population, we found increased risk for suicide for males from WCS Africa, NEH Africa and CES Europe. The number of suicide deaths in females was too small to be able to draw any conclusions. No suicide deaths were recorded in asylum seekers under the age of 15 (data not shown).

**Table 1:** Suicide death rates in asylum seekers by sex, age, region of origin and rate ratios for asylum seekers in comparison with the general population of the Netherlands

Sex	Subgroup	Number of person years	Number of suicide deaths	Suicide mortality rate/100 000/year	Mortality rate ratio*	95% CI
**Males**						
	Netherlands	39 068 490	6131	15.7	1	
	Asylum seekers	125 026	32	25.6	2.00*	1.37-2.83
**Asylum seekers by age group**						
	15-24	44 171	8	18.1	2.33	1.16-4.70
	25-34	42 459	13	30.6	2.32	1.34-4.01
	> = 35	38 396	11	28.6	1.58	0.88-2.86
**Asylum seekers by region#**						
	ME/SW Asia	40 278	7	17.4	1.28*	0.51-2.63
	WCS Africa	31 205	8	25.6	2.36*	1.01-4.63
	NEH Africa	14 284	6	42.0	3.32*	1.21-7.19
	CES Europe	28 165	9	32.0	2.31*	1.06-4.38
**Females‡**						
	Netherlands	40 509 314	2854	7.0	1	
	Asylum seekers	74 916	3	4.0	0.73*	0.15-2.07

# numbers do not add up to total as not all regions are presented ‡ No further breakdown because of small number of cases * rate ratios standardized for age

There were 290 cases of suicidal behaviour treated in hospital (table 2). Hospital-treated suicidal behaviour was more common in female asylum seekers than in male asylum seekers (RR = 1.58; 95% CI = 1.25-1.99). Compared to the population of The Hague, male and female asylum seekers from CES Europe and ME/SW Asia were at increased risk of hospital-treated suicidal behaviour; asylum seekers from NEH and WCS Africa were at lower risk of hospital-treated suicidal behaviour (table 2). In the age group < 15 years we recorded 11 acts of hospital-treated suicidal behaviour in girls but none in boys (data not shown).

**Table 2:** Hospital-treated suicidal behaviour rates by sex, age, region of origin and rate ratios for asylum seekers in comparison with the population of The Hague

Sex	Subgroup	Number of person years	Number of hospital-treated suicidal behaviour cases#	Hospital-treated suicidal behaviour rate/100 000/year	Hospital-treated suicidal behaviour rate ratio*	95% CI
**Males**						
	The Hague	558 762	489	87.5	1	
	Asylum seekers	125 026	149	119.2	1.42*	1.20-1.66
**Asylum seekers by age group**						
	15-24	44 171	48	108.7	1.49	1.02-2.17
	25-34	42 459	39	91.9	1.02	0.71-1.46
	> = 35	38 396	58	151.1	1.68	1.27-2.22
**Asylum seekers by region**						
	ME/SW Asia	40 278	55	136.6	1.60*	1.20-2.08
	WCS Africa	31 205	22	70.5	0.88*	0.55-1.32
	NEH Africa	14 284	5	35.0	0.41*	0.13-0.96
	CES Europe	28 165	57	202.4	2.37*	1.79-3.07
**Females**						
	The Hague	595 215	947	159.1	1	
	Asylum seekers	74 916	141	188.2	1.00*	0.84-1.18
**Asylum seekers by age group**						
	15-24	22 655	34	150.1	0.51	0.36-0.73
	25-34	24 384	46	188.6	1.18	0.86-1.63
	> = 35	27 877	56	200.9	1.57	1.19-2.07
**Asylum seekers by region**						
	ME/SW Asia	21 894	55	251.2	1.40*	1.06-1.82
	WCS Africa	13 570	5	36.8	0.17*	0.05-0.38
	NEH Africa	8041	3	37.3	0.19*	0.04-0.55
	CES Europe	23 992	61	254.3	1.44*	1.10-1.85

# numbers do not add up to totals as age is not available for all cases and not all regions are presented * rate ratios standardized for age

### Factors that could guide prevention

#### Mental health problems

In nearly 80% of cases of hospital-treated suicidal behaviour, the notifying health professional was aware of the existence of mental health problems and nearly three quarters of the asylum seekers concerned were receiving some form of mental health treatment prior to the hospital-treated suicidal behaviour (table 3). The table shows no significant differences between male and female cases with respect to health professionals' knowledge about their mental health problems, but mental health treatment was 20% more common in females. The prevalence of mental health treatment seems to be lower for asylum seekers originating from Africa compared with people from other regions, while only small differences were found in the known existence of mental health problems (table 3).

**Table 3:** Distribution of mental health problems and mental health treatment among hospital-treated suicidal behaviour cases

	% of suicidal behaviour cases for which health staff knew of the existence of mental health problems prior to the reported suicidal behaviour (absolute number of cases)#	Prevalence rate ratio (95% CI)	% of suicidal behaviour cases with known mental health problems that received mental health treatment prior to the reported suicidal behaviour (absolute number of cases)‡	Treatment rate ratio (95% CI)
**Total**	78.7 (222)		73.0 (162)	
**Sex**				
Male	74.5 (108)	1	66.7 (72)	1
Female	83.2 (114)	1.12 (0.99-1.26)	78.9 (90)	1.18 (1.01-1.39)
**Age group**				
15-24	64.6 (53)	1	64.2 (34)	1
25-34	82.4 (70)	1.27 (1.06-1.54)	72.9 (51)	1.14 (0.98-1.45)
> = 35	85.2 (98)	1.32 (1.10-1.57)	77.6 (76)	1.21 (0.96-1.52)
**Region of origin**				
ME/SW Asia	78.0 (85)	1	72.9 (62)	1
WCS Africa	63.0 (17)	0.86 (0.58-1.53)	29.4 (5)	0.41 (0.32-2.05)
NEH Africa	75.0 (6)	0.96 (0.37-1.94)	33.3 (2)	0.38 (0.11-2.78)
CES Europe	83.8 (98)	1.06 (0.81-1.21)	80.6 (79)	1.14 (0.79-1.23)

# number of hospital-treated suicidal behaviour cases for which health staff were aware of the existence of mental health problems;

‡ number of hospital-treated suicidal behaviour cases for which health staff were aware of the existence of mental health problems and who were receiving mental health treatment

#### Stressors

The most frequently reported stressor for hospital-treated suicidal behaviour was the asylum procedure (30.4%). The other stressors were: relationship issues (21.2%), loss of a family member (13.1%), transfer between centres (9.2%), substance abuse (9.2%) and living conditions (3.9%).

#### Methods used

The distribution of methods used differed considerably between cases of death from suicide and hospital-treated suicidal behaviour (table 4). In suicide deaths, hanging was the most common method used and in hospital-treated suicidal behaviour cases, this was poisoning with drugs.

**Table 4:** Distribution of methods used in suicide death and hospital-treated suicidal behaviour cases

Method used	Suicide death cases (%)*	Hospital-treated suicidal behaviour cases (%)*
Hanging	5 (25.0)	8 (2.9)
Poisoning by drugs	3 (15.0)	203 (72.8)
Moving object	3 (15.0)	3 (1.1)
Poisoning by other means	2 (10.0)	12 (4.3)
Drowning	2 (10.0)	2 (0.7)
Jumping	2 (10.0)	6 (2.2)
Cutting	2 (10.0)	39 (14.0)
Burning	1 (5.0)	6 (2.2)

* as a percentage of the cases for which a method was reported

## Discussion

This study is, to our knowledge, unique in the length of the study period, the number of cases observed and the combination of suicide death and suicidal behaviour data. The suicide death rate was high in male asylum seekers compared with the reference population, whereas no conclusion could be drawn for females due to the small number of cases. The hospital-treated suicidal behaviour incidence was not homogenously distributed in the asylum seeker population. In several subgroups the rates were higher than in the reference population, but lower rates were also found. Among African asylum seekers, male suicide mortality was high, but male and female hospital-treated suicidal behaviour rates were low. Among Europeans, the suicide mortality rate was low for males and hospital-treated suicidal behaviour rates were high for both sexes. For Middle East/South West Asians, only hospital-treated suicidal behaviour rates were increased. Health professionals were aware of mental health problems existing prior to the hospital-treated suicidal behaviour in nearly 80% of the cases.

### Methodological considerations

All suicide death notifications contained clear case descriptions, so we do not expect any overestimation with regard to suicide death. It is possible, as in other studies of this kind, that some suicide deaths have not been included because they may have been coded under other external causes of death [18]. Cases of death in asylum seekers for which no cause has been reported can also hide suicides. However, because of the huge impact of a suicide death in an asylum seeker centre, we assume that virtually all cases considered suicide deaths were reported. For hospital-treated suicidal behaviour, on the other hand, we expect that our results are an underestimation as some of this behaviour may not have come to the attention of MOA staff. Moreover, the reporting may have been incomplete. But due to the continuous presence of medical staff at the asylum centres, cases may have come to their knowledge that might have been missed in the general population.

The results of this study should be interpreted with caution because of the small number of cases, particularly for suicide death. No conclusion could be drawn with respect to suicide mortality among females because of the small number of cases. Comparison with data from other studies has to be done cautiously because of differences in data sources, particularly for the hospital-treated suicidal behaviour rate. The use of suicidal behaviour reference data for the city of The Hague may have influenced our results. The incidence rate in The Hague is 10-20% higher than estimates for the Netherlands [21]. This implies that for the subgroups with increased hospital-treated suicidal behaviour rates, the difference with the general population of the Netherlands would be somewhat larger. For the African groups, for whom we observed lower rates, the difference would be somewhat smaller, but would still remain.

### Interpretation of results

The current study suggests that suicide death is higher in male asylum seekers than in the general male population of the Netherlands. The incidence was higher than in the reference population for several regions of origin, despite possible underreporting. As in many populations, in asylum seekers suicide was more prevalent in men and hospital-treated suicidal behaviour more prevalent in women [5]. The male to female ratio for the number of suicide deaths in this study (10:1), however, was higher than generally found (between 3:1 and 7.5:1) [5]. Various hypothesises can be formulated for this gender difference. The higher risk for males could be related to the lower use of mental health services. Additional explanations are that males are at greater risk if they are forced to return to their country of origin, the supposed higher pressure to succeed, more negative consequences of not being allowed to work and a higher prevalence of drug use [25,26]. Protective factors for women could be having children to care for and stronger social networks [26]. The gender differences for hospital-treated suicidal behaviour varies between regions of origin, reflecting the general fact that the gender difference paradox in suicidal behaviour is not constant across countries [27].

The regional differences found in hospital-treated suicidal behaviour rates are fairly consistent with data from Sweden, where the rates showed considerable differences between countries and regions of origin, with the lowest risk among Africans [7]. In general, rates in immigrants tend to co-vary with rates in the country of birth [3]. It is uncertain whether the differences found between regions reflect suicide death and hospital-treated suicidal behaviour rates in countries of origin, as we had to group countries together and because suicide statistics for countries of origin were either unavailable or outdated [28].

The majority of asylum seekers with hospital-treated suicidal behaviour were known to suffer from mental health problems. A recent review states that evidence on a relationship between PTSD and suicide or suicidal behaviour among refugees is limited [29]. In a retrospective case-control study in asylum seekers in the Netherlands, including 40 suicide deaths and 40 matched controls, PTSD was not associated with suicide, and mental health treatment in the home country was more prevalent in suicide cases than controls (unpublished study by Koeman M and Kerkhof AJFM, 2004).

The much lower treatment rate of mental health problems in people with hospital-treated suicidal behaviour from Africa, compared with people from ME/SW Asian and CES European origin, is a concern. Low mental health treatment rates in African asylum seekers and refugees have been reported in other studies in the Netherlands and the UK [30,31].

Hanging was the most common method in deaths from suicide in asylum seekers; it is also the most common method in deaths from suicide in the general population, according to a European study into suicide methods [32]. However, the proportion of asylum seekers that used hanging (25%) was lower than in the European study (50%). This might be related to the housing situation; asylum seekers share their room with others and have little privacy. In cases of hospital-treated suicidal behaviour, poisoning by drugs was the most common method and cutting the second most common. This pattern is similar to that observed in both migrants and non-migrants in the WHO/EURO multi-centre study on suicidal behaviour [33].

Relationship issues, loss of family members, and other stressful life events are associated with suicidal behaviour in the general population [3,5], and are also commonly reported in asylum seekers.

The asylum procedure, however, is a stressor specific for this population. Asylum procedure recognition rates may well influence suicide death and suicidal behaviour rates. As recognition rates and other factors, such as composition of the asylum population, reception conditions and accessibility of health care for asylum seekers vary between countries and over time [34,35], we expect that suicide death rates and suicidal behaviour will vary between host countries and may change over time. Comparative research in various host countries, including qualitative research, is required to assess how national asylum policies influence the rate of suicide and suicidal behaviour in asylum seekers and which preventive interventions are effective.

Training physicians to recognize and treat depression and suicidal behaviour has already shown impressive effects in reducing suicide death rates in general populations and might contribute to reduction in asylum seekers as well [17]. In parallel with suicide prevention in prisons [36], health professionals and reception centre personnel who are working with asylum seekers should be trained to recognize suicide ideation and to take appropriate action.

## Conclusions

This study suggests that male asylum seekers are at increased risk of death from suicide in comparison with the population of the Netherlands. No conclusion could be drawn for females due to the small number of cases. For males and females the incidence of hospital-treated suicidal behaviour was higher for asylum seekers from ME/SW Asia and CES Europe than in the reference population and lower for asylum seekers from the African sub regions. The majority of people with hospital-treated suicidal behaviour from ME/SW Asia and CES Europe had received some form of mental health treatment prior to the hospital-treated suicidal behaviour. For asylum seekers from Africa the rate of mental health treatment seemed to be lower. On the basis of this study we conclude that targeted prevention of suicide death and suicidal behaviour in asylum seekers is indicated.

Ethics Committee approval was not necessary as only anonymous data were used that were collected in the light of regular health service provision. The use of anonymous data for epidemiological studies is, in line with the medical ethical standards in the Netherlands, included in the privacy statement of the Community Health Services for Asylum Seekers.

## Competing interests

The authors declare that they have no competing interests.

## Authors' contributions

SG and AK designed the study; all authors critically contributed to the interpretation of the data and to the content of the article; all authors approved the final version.

## Pre-publication history

The pre-publication history for this paper can be accessed here:

http://www.biomedcentral.com/1471-2458/11/484/prepub
